# The Challenge of Measuring UK Wealth Inequality in the 2000s†

**DOI:** 10.1111/j.1475-5890.2016.12084

**Published:** 2016-03-31

**Authors:** Facundo Alvaredo, Anthony B. Atkinson, Salvatore Morelli

**Affiliations:** ^1^ Paris School of Economics; INET at the Oxford Martin School; Conicet; ^2^ Nuffield College; London School of Economics; INET at the Oxford Martin School; ^3^ CSEF – University of Naples ‘Federico II’; INET at the Oxford Martin School

**Keywords:** wealth inequality, estate multiplier, investment income, D3, H2

## Abstract

The concentration of personal wealth is now receiving a great deal of attention – after having been neglected for many years. One reason is the growing recognition that, in seeking explanations for rising income inequality, we need to look not only at wages and earned income but also at income from capital, particularly at the top of the distribution. In this paper, we use evidence from existing data sources to attempt to answer three questions: (i) What is the share of total personal wealth that is owned by the top 1 per cent, or the top 0.1 per cent? (ii) Is wealth much more unequally distributed than income? (iii) Is the concentration of wealth at the top increasing over time? The main conclusion of the paper is that the evidence about the UK concentration of wealth post‐2000 is seriously incomplete and significant investment in a variety of sources is necessary if we are to provide satisfactory answers to the three questions.

## Policy points

- The evidence about the UK distribution of wealth post‐2000 is seriously incomplete.
- Significant investment in statistics is necessary if we are to be able to draw firm conclusions about the extent of wealth concentration.

## I. Wealth inequality under the spotlight

The distribution of personal wealth is now receiving a great deal of attention – after having been neglected for many years. One reason is the growing recognition that, in seeking explanations for rising income inequality, we need to look not only at wages and earned income but also at income from capital. Income from interest, from dividends and from rents represents a minority of total personal income, but it is nonetheless a significant part, in particular, at the top of the distribution. Moreover, viewed from the side of National Accounts, the share of income from capital and rents has been increasing in recent decades. In many OECD countries, the ratio of total personal wealth to total personal income has been rising. One consequence is that the role of inherited wealth – declining for much of the 20^th^ century – has, in a number of countries, begun to acquire greater significance.

The recent attention to the distribution of wealth has led to evidence being sought on several key questions. The first is the extent of concentration of wealth at the top. What is the share of total personal wealth that is owned by the top 1 per cent, or by even smaller groups such as the top 0.1 per cent? The second question is whether wealth is much more unequally distributed than income. The OECD report, *In It Together*, stresses that ‘household wealth – in particular financial assets – is much more unequally distributed than income’.^1^ The third key question is whether wealth inequality is increasing over time. The OECD report says of the UK that ‘the financial crisis has exacerbated the concentration of wealth at the top’.^2^ How far is wealth inequality increasing?

In this paper, we examine the evidence on these three questions for the United Kingdom, focusing on the period from 2000 onwards. The first prerequisite is to consider the range of sources of evidence about wealth‐holding. There has been disagreement in the UK literature about the level and trend in the distribution of wealth, and this disagreement stems in part from the use of different sources. Section I. summarises the main ‘windows’ through which we can observe the distribution of wealth in the UK, drawing attention to their strengths and weaknesses. The second prerequisite is to clarify definitions. There is no such thing as ‘the’ distribution of wealth. A figure for the share of the top 1 per cent could relate to the top 1 per cent of households, or of families, or of individuals. The share could relate to wealth excluding or including pension rights; the pension rights could include state pensions or be limited to private pensions. The 1 per cent could be limited to residents or could include those non‐domiciled. Section 5. of the paper sets out some of the key definitional issues. Having cleared the ground, we examine in Section 3. the light that existing evidence casts on the answers to the three key questions posed in the previous paragraph. This examination leads us to identify important ways in which there needs to be investment in improving the informational base about wealth‐holding in the UK. The main conclusions are summarised in Section 3..

## II. Different windows on wealth

There are four main potential sources of evidence about the distribution of personal wealth in the UK:

- administrative (tax) data on estates at death, which indirectly provide evidence about the wealth of the living, by applying (the inverse of) mortality multipliers differentiated by age, sex and wealth class;
- administrative (tax) data on investment income, which indirectly provide evidence about the wealth of the living, by applying yield multipliers;
- household surveys of personal wealth, such as the Wealth and Assets Survey (WAS) conducted by the Office for National Statistics (ONS);
- lists of large wealth‐holders, such as the Sunday Times ‘Rich List’, which has been compiled by Philip Beresford in the UK, and the Forbes List of Billionaires.^3^

There are in addition synthetic estimates that draw on two or more sources, such as those of Credit Suisse Research Institute (2014), which combines household survey data from the WAS with the *number* of Forbes billionaires, and Vermeulen (2015), who combines extreme observations on the *number* of billionaires as well as their *wealth* from the Forbes List with the WAS data. In all cases, the evidence about the *distribution* of wealth has to be considered in relation to the external control totals for population, based on demographic data, and for total personal wealth.

Each of the sources is considered below, where we summarise the methods and their main strengths and weaknesses.

### 1. The multiplied estate data

Estimates of the distribution of wealth based on administrative data from the taxation of the estates of those dying in a particular year are reached by applying the estate multiplier method. Estimates of the estate distribution are first obtained from a sample of the estates submitting an inheritance tax return.^4^ Subsequently, the method considers the grossed‐up population of decedents as a sample of the living population. The death rate, however, is clearly not random, as it varies substantially across age, gender, and social or wealth class, etc. One can nonetheless define death as ‘random’ within each specific age, gender, marital status, social or wealth class cell, and take each cell‐specific mortality rate as the ‘sampling rate’. Their inverse (‘estate multiplier’) can then be used to re‐weight the observations for decedents in order to obtain the distribution for the living population. Additional adjustments have to be made in order to control for individuals not covered by the estate tax statistics and total assets not represented in the estate data.

These data on estates at death have long been used for economic research in the UK. Initially, they were employed to make estimates of total personal wealth. Baxter (1869) estimated the total wealth on the basis of the Probate Duty data, applying a multiplier of 30, which he took to be the cycle for each devolution of property. In terms of the distribution, such a single multiplier, referred to as a ‘unity multiplier’, means that this method yields estimates of the distribution of estates, not of wealth. It takes no account of the differential rates of death by wealth class. Following the proposal of Coghlan (in the discussion of Harris and Lake (1906)), Mallet (1908) applied to each estate a multiplier related to age at death. These ‘general mortality multipliers’ were subsequently refined in Mallet and Strutt (1915) to apply ‘social class multipliers’ allowing for the lower mortality of the upper and middle classes. Differentiation was also made later on the basis of gender. For many years, this has formed the basis for estate‐based estimates of the distribution of wealth in the UK.

Applying multipliers yields estimates of the number of individuals owning wealth in particular ranges and the amounts of their wealth. The next step is to relate the numbers and amounts to external control totals. In the latter case, the totals come from elements in the national balance sheets. This method was developed in Atkinson and Harrison (1978) and by the Inland Revenue in its (revised) Series C introduced to cover the period from 1976. Series C was published on an annual basis until 2005. A new methodology has since been introduced by HM Revenue & Customs (which has replaced the Inland Revenue), with estimates being produced for three‐year averages 2001–03, 2005–07 and 2008–10.^5^ The details of the change are discussed further in Section 3..1.

The estate‐based estimates have evident shortcomings. The first, and most obvious, is that the estate data as such do not cover the rights to occupational or state pensions.^6^ The second, equally obvious, is that the degree of concentration of wealth is likely to be understated on account of tax avoidance and evasion. Estate planning is certainly an effective way to reduce tax liabilities at death. In the UK, for instance, assets given away at least seven years before death are not subject to estate taxation. In statistical terms, this problem is mitigated by the fact that the recipients are also subject to the risk of dying, and their multiplied‐up wealth appears in the estimated wealth distribution. However, the donors are likely to be unrepresentative of their class (being less healthy) so that the mitigation is only partial.^7^ A second source of avoidance is provided by trusts (mainly discretionary trusts). Although the official Series C attempted to make allowance for excluded wealth in trusts, these adjustments were based on limited and increasingly dated information.

Moreover, the validity of the estate multiplier method depends on the estate multipliers. The HMRC wealth model approximates the mortality risk of wealthy individuals with that of individuals who are owner‐occupiers taken from the ONS Longitudinal Study of social class and occupational mobility. This model was recently updated using the English Longitudinal Study of Ageing (ELSA) to better capture the relationship between housing wealth and mortality.^8^ Similarly, Kopczuk and Saez (2004) use the mortality of US college‐educated individuals as a proxy for that of wealthy individuals. However, Saez and Zucman (2014) report that this may not be a good approximation of mortality for wealthier people (above the 90^th^ percentile of the wealth distribution), whose mortality rate is considerably lower. They argue that this mortality gap has been increasing over time in the US, biasing downward the evolution of the estate‐based wealth shares. The estate multiplier approach would clearly benefit from a fresh systematic investigation of mortality risk within the population according to social classes and income and wealth levels.

### 2. The multiplied investment income data

The investment income method has been applied to the UK by Atkinson and Harrison (1974 and 1978, ch. 7), building on the work of Barna (1945) and Stark (1972). The underlying method has been described by Saez and Zucman (2014, p. 1) in their recent paper on the US as follows:

Starting with the capital income reported by individuals to the Internal Revenue Service—which is broken down into many categories: dividends, interest, rents, profits, mortgage payments, etc.—for each asset class we compute a capitalization factor that maps the total flow of tax income to the total amount of wealth recorded in the Flow of Funds. We then combine individual incomes and aggregate capitalization factors by assuming that within a given asset class the capitalization factor is the same for everybody. For example, if the ratio of Flow of Funds fixed income claims to tax reported interest income is 50, then $50,000 in fixed income claims is attributed to an individual reporting $1,000 in interest.

They use the income tax data and the national balance sheets (Flow of Funds). This may be contrasted with the ‘hybrid’ investment income method used by Atkinson and Harrison, where the yields are taken from external sources and weighted using asset composition data from the estate‐based wealth estimates, leading to a single capitalisation factor applied to total investment income reported in the Survey of Personal Incomes (SPI), based on income tax returns. The adoption of this hybrid approach reflected the fact that the income data in the UK were only tabulated according to broad categories.^9^ It is also the case that, where the estate‐based asset composition data include assets that do not generate income taxable under the income tax, these can be allowed for in calculating the overall multiplier. Such assets include owner‐occupied houses, non‐interest‐bearing bank accounts, non‐taxable fixed‐income claims, durable goods and collectibles. In contrast, Saez and Zucman make allowance for such assets making use of additional data sources: surveys, property tax records, etc; and they are also able to attach estimates of funded pension wealth.

The theoretical basis for the investment income method and the potential bias in the estimation of wealth inequality are set out in Atkinson and Harrison (1978, ch. 7 and appendix VII), where two main sources of error are identified: the variation with the level of wealth of the rates of return to individual asset types, and the variation in the rate of return for a given asset and wealth level (idiosyncratic returns). The US estimates of Saez and Zucman (2014) represent an advance in that they employ data from foundations to demonstrate that returns are flat within asset classes (the overall yield rises with wealth on account of asset composition). In the case of the second source of error, the authors argue from an illustrative calculation that ‘idiosyncratic returns cannot create much bias’ (p. 16). The discussion in Atkinson and Harrison (1978) is more cautious, concluding that the upward bias in the measurement of wealth inequality ‘is large enough to be taken seriously but not sufficient to discredit the investment income method’ (p. 199).

The investment income method has considerable advantages in that the underlying data relate to the living population and the method does not depend on assumptions about the differential mortality rates by wealth classes. Estimates employing the hybrid investment income method were made by Atkinson and Harrison (1978) for 1968–69 and 1972–73. Today, however, it does not seem possible to satisfactorily apply the method using the currently available data. The SPI micro data available to public users only provide four variables (aggregating many different types of capital incomes): (i) dividends; (ii) income from property; (iii) net interest from UK banks, building societies and other deposit takers; and (iv) other investment income. In order to apply the full investment income method, a more detailed version of the SPI micro data would be necessary. The information contained in the internal SPI looks more promising, but at the moment of writing we have not yet obtained effective access to the micro data.^10^

The application of the hybrid method, as in Atkinson and Harrison (1978), could be contemplated, but this requires a detailed breakdown of wealth by asset types and wealth ranges. The published information for years 2000 onwards^11^ only gives six categories of assets and two of liabilities. Again, to apply the investment income method, more detailed information is required.

In our view, the investment income method should certainly be explored further, but for this it is necessary that the underlying data be available in a more detailed form.

### 3. Household surveys

Household surveys are a quite different source of data, unaffected by problems of tax avoidance and tax evasion because unrelated to the operation of the tax system, and able to furnish information about pension entitlements. These surveys date back in the UK to the Oxford Savings Surveys in the 1950s.^12^ In the 1970s, the Royal Commission on the Distribution of Income and Wealth investigated the possible role of sample surveys of wealth‐holding, commissioning two small pilot surveys, but concluded that the results, ‘notably the particularly low response rate (around 50 per cent)’, did not justify the launching of a full‐scale survey.^13^

More recently, attitudes towards household surveys have changed. The British Household Panel Survey (BHPS) began collecting data on financial wealth in 1995. In 2000, the Office for National Statistics began to plan the longitudinal Wealth and Assets Survey (WAS), which was launched in 2006, funded by a consortium which also included (in 2012) the Department for Work and Pensions, HMRC, the Financial Conduct Authority and the Scottish Government.^14^ The first WAS spanned the period 2006–08, and subsequent waves have covered 2008–10 (Wave 2), 2010–12 (Wave 3) and 2012–14 (Wave 4, full results not yet published), covering only Great Britain.

**Figure 1:**
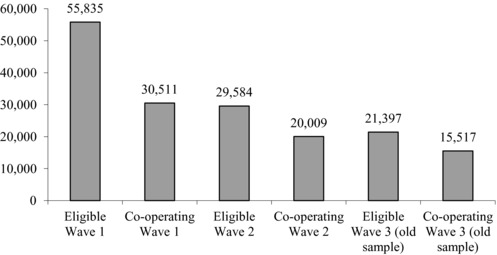
Eligible and cooperating households in each WAS wave *Source*: Data provided by Office for National Statistics.

Does the renewed interest in household survey data on wealth reflect a resolution of the problem of low response rates? This does not appear to be the case. Wave 1 of WAS in 2006–08 achieved a response rate of 54.6 per cent – similar to that found in the 1970s. Since the WAS is a longitudinal survey, the calculation of the combined response rate over successive waves is not straightforward, as the ONS attempts to re‐contact previous wave non‐contacts and movers (household splitting) between waves. Figure 1 shows the absolute number of households eligible at each stage and the number cooperating. Waves 2 and 3 achieved higher response rates among those eligible, but this still left a final total of only 15,517 households, compared with an initial eligible sample of 55,835 in Wave 1. Wave 3 included a new ‘booster’ sample, with a response rate of 50.8 per cent.

A low rate of response does not necessarily imply that the results on wealth shares are biased. On the other hand, there are a priori reasons to expect there to be differential non‐response by wealth classes. The feasibility studies in the 1970s found that ‘the indications were that non‐response would be higher among those groups with higher incomes and substantial investment income’.^15^ In order to mitigate this effect, the WAS made use of information available from the income tax records to flag addresses where at least one person was likely to have total financial wealth above a certain threshold, and these flagged addresses had a higher (two‐and‐a‐half or three times) chance of selection.^16^ However, the evidence gathered in Vermeulen (2015) suggests that the oversampling strategy has not been very effective. There were also problems of incomplete response. In the case of business assets, ‘a high percentage of those who said they held business assets failed to provide an estimate of the value of such assets’.^17^ This led to business assets being excluded from the estimates of total wealth. This omission is likely to be particularly important in the upper wealth ranges.

The issues of non‐response and under‐reporting at the top mean, in our view, that the Wealth and Assets Survey – valuable as it is in covering the majority of the population – cannot, on its own, provide a fully satisfactory representation of the upper tail of the UK wealth distribution.

### 4. The Rich Lists

Since 1989, the *Sunday Times* has published annually in April a ‘Rich List’ of the wealthiest people or families in Great Britain. The lists, compiled by Philip Beresford, appear as a supplement to the newspaper, and on occasion in extended book form.^18^ The methods used in constructing the lists are set out in ‘Rules of engagement’.^19^ The description emphasises that the estimates are ‘the minimum wealth … the actual size of their fortunes may be much larger’. The construction of the list draws on a wide range of public information, coming from a variety of sources. The estimates relate to identifiable wealth, such as land, property, art, or significant shares in publicly‐quoted companies, and in recent years have paid particular attention to liabilities (for example, where shares are used as collateral for loans).

UK top wealth‐holders are also included in the global Forbes List of (Dollar) Billionaires, published annually by the business magazine since 1987. The list is compiled by reporters who ‘meet with the list candidates and their handlers and interview employees, rivals, attorneys and securities analysts. … We do attempt to vet these numbers with all billionaires. Some cooperate, others don't’.^20^ Nonetheless, it is not easy to validate the information.

In summary, the Rich Lists provide valuable insight into the upper tail of the wealth distribution, but it is not easy to assess their representativeness.

### 5. Total personal wealth

The shares of top wealth‐holders depend on the control total for personal wealth. In the early part of the period with which we are concerned here, HMRC provided a reconciliation of the wealth totals that is of central importance in understanding the estimates of wealth shares.^21^ This is particularly useful to control for the individuals as well as the total assets not represented within the estate data (‘excluded wealth’).

The reconciliation begins with the total net wealth identified in the multiplied‐up estate data (‘identified wealth’), which was £3,432 billion in 2005, as shown in the online appendix. The first stage involves adjustment for under‐recording and differences in valuation in the estate data (for example, replacing the maturity value of a life assurance policy by its equity value). This increases the total in 2005 to £4,097 billion. To this is added the estimated value of the so‐called ‘excluded wealth’ – namely, that wealth not subject to estate taxation as well as the wealth of those not covered by the estate data. The so‐called ‘excluded wealth’ includes estimates of joint properties, small properties and trusts. The resulting total is £5,005 billion in 2005, and this is defined as ‘Series C marketable wealth’. The total is 46 per cent higher than ‘identified wealth’.

The Series C total marketable wealth may be compared with the total sector (S.14 and S.15 combined) wealth in the National Accounts balance sheets. There are significant definitional differences. The first is that the National Accounts combine households with non‐profit institutions serving households (NPISH); the second is that the National Accounts balance sheets are defined on an end‐of‐year basis. The most important difference, however, is the inclusion in the National Accounts total of the value of funded pension rights (£1,213 billion in 2005). The aggregate value of all pension rights, funded and unfunded, occupational and state, is given as £2,999 billion in 2005.

It is evident that the adjustments to the estate data, and whether or not pension wealth is included, make a significant difference to the control totals employed.

## III. Inequality of what among whom?

The paper is concerned with the distribution of personal wealth, by which we mean the value of the total assets owned (directly or indirectly) by individuals, net of their debts. Assets include financial assets, such as bank accounts, stocks or bonds, and real assets, such as houses, business assets and consumer durables. As defined here, it does not include ‘human capital’ (the capitalised value of future earnings).

The implementation of this concept does, however, raise a number of definitional issues, and these are resolved in different ways in different sources of evidence.

### 1. Geographical scope

First, there is the geographical scope. The estimates discussed here relate either to the United Kingdom (tax‐based estimates) or to Great Britain (the WAS household survey), the latter excluding Northern Ireland. Northern Ireland accounts for 2.8 per cent of the UK total resident population. However, the Sunday Times Rich List has a different approach. It includes people who live and work in Britain, and people who are married to Britons, who have strong links with Britain, who have estates and other assets there, or who have backed British political parties, British institutions and British charities. It includes British citizens abroad. The population represented is therefore more extensive than that in the estate‐based estimates, or investment income data, or the WAS household surveys.

### 2. Unit of analysis

The unit of analysis in the case of the estate‐based estimates and the investment‐income‐based estimates is the individual.^22^ Estates are naturally recorded on the death of an individual. Since 1990, the income tax has been levied on an individual basis, and hence the investment income data take this form. In contrast, the WAS survey data relate to the total wealth of the household, defined as a person or a group of people (family members and non‐relatives) living together in the same dwelling.^23^ In the case of the Rich Lists, the unit may be more extensive than the household. For example, in the 2014 Sunday Times list, the top entry was the Hinduja brothers; third was Lakshmi Mittal and family, which includes his son and daughter; the wealth of number 11 includes that of Galen Weston, his wife and his nephew, George Weston. There are often multiple generations, such as number 19 (Earl Cadogan and his son, Viscount Chelsea).

What difference does the unit of analysis make to the estimated wealth shares? How can we compare the estate‐based estimates of individual wealth with the household wealth estimates in the WAS? If we treat all units as weighted equally (so no account is taken of household size), then the control total for households is smaller than that for individuals (by a factor 1/*h*, less than 1, where *h* is the average number of adults per household). In 2010 in the UK, the value of *h* is close to 2, and we take that value in the illustrative examples below. The impact of moving from an individual to a household basis depends on the joint distribution of wealth. Suppose first that in the top 1 per cent of individuals each person is married to someone with equal wealth. They then constitute the top 1 per cent of households (since *h* = 2) and have the same share of total wealth. On the other hand, to the extent that the top 1 per cent marry out of that group, the household‐based share of total wealth is reduced. Similarly, if the top 1 per cent of individuals are all single, then they account for 2 per cent of total households, and the share of the top 1 per cent is reduced, compared with that measured on an individual basis. The calculations in Atkinson and Harrison (1978, p. 248) suggest that, in the limiting cases of all single or of rich married to poor, the share of the top 1 per cent could be reduced by 4 to 5 percentage points when moving from the individual to the household distribution. In practice, the household‐based estimates are likely to be lower but by less than this amount.

### 3. Method of valuation

A third set of definitional issues concerns the method of valuation, a topic that is often taken for granted. As the ONS says of national balance sheets,^24^ the wealth figures are taken to represent the ‘market value of the financial and non‐financial assets’, but the application of the market value approach raises a number of issues. Life assurance policies provide an illustration. This asset changes value on death: the maturity value recorded in the estate exceeds the value to the person alive. For this reason, HMRC in its Series C made adjustments. But the market value, in terms of what the policy would fetch if surrendered, falls short of the continuing value to the person. Atkinson and Harrison (1978, p. 5) distinguish between ‘realisation’ and ‘going concern’ valuations. Interpreted as what a person could realise by the sale of all assets, net of liabilities, the former coincides in principle – with exceptions such as life policies – with the value placed on an estate at death. The going‐concern valuation, however, could well be considerably higher. That there can be a significant difference may be seen from the example of household contents (durables, furniture, etc.), where the price obtained on sale is likely to fall considerably short of the value to a continuing household (or the replacement cost). A less common, but important, example of differences between realisation and going‐concern valuations is that of family businesses. Finally, there is the case of pension rights, where the realisation value may be zero, but they are of considerable value to a living person. The standard approach to handling these differences is by the exclusion or inclusion of classes of assets. The current HMRC estate‐based estimates exclude pension rights (private and state). The WAS estimates both include and exclude pension rights. The WAS estimates also exclude business assets. However, it seems preferable to adopt explicitly either a realisation or a going‐concern basis.

## IV. Wealth shares in the UK since 2000

We discuss in turn the different sources of evidence about the distribution of wealth in the UK and the conclusions that can be drawn about the three questions posed at the start of the paper. As noted above, no results are given using the investment income method, since we do not yet have access to the necessary data.

### 1. Estate‐data‐based estimates

**Figure 2:**
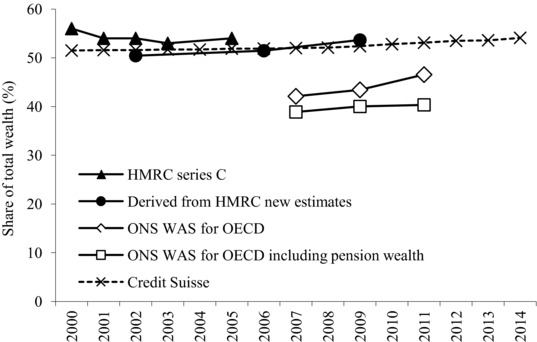
Estimates of the top 10 per cent wealth shares since 2000 *Note*: The WAS estimates relate to households and to Great Britain.

**Figure 3:**
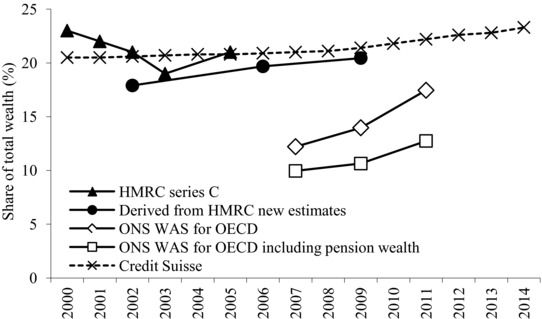
Estimates of the top 1 per cent wealth shares since 2000 *Note*: The WAS estimates relate to households and to Great Britain.

We begin with HMRC Series C, which covers the years from 2000 to 2005 (excluding 2004). The shares of the top 10 per cent, 5 per cent and 1 per cent are shown in Figures 2 and 3 and Table 1. The published data also include the share of the top 50 per cent, top 25 per cent and top 2 per cent; they do not break down the top 1 per cent.

**Table 1:** Estimates of top wealth shares

*Per cent*																		
	HMRC Series C			Derived from HMRC new series			ONS WAS for OECD			ONS WAS for OECD incl. pension wealth			Credit Suisse		Vermeulen			
	*Top 10%*	*Top 5%*	*Top 1%*	*Top 10%*	*Top 5%*	*Top 1%*	*Top 10%*	*Top 5%*	*Top 1%*	*Top 10%*	*Top 5%*	*Top 1%*	*Top 10%*	*Top 1%*	*Top 5%, lower bound*	*Top 5%, upper bound*	*Top 1%, lower bound*	*Top 1%, upper bound*
2000	56.0	44.0	23.0										51.5	20.5				
2001	54.0	41.0	22.0										51.6	20.5				
2002	54.0	41.0	21.0	50.4	37.5	17.9							51.6	20.6				
2003	53.0	40.0	19.0										51.7	20.7				
2004													51.7	20.8				
2005	54.0	40.0	21.0										51.9	20.8				
2006				51.5	38.2	19.7							51.9	20.9				
2007							42.1	29.4	12.2	38.9	26.3	10.0	52.0	21.0				
2008													52.1	21.1				
2009				53.7	40.1	20.4	43.4	30.8	14.0	40.0	26.9	10.6	52.4	21.4	31.0	35.0	14.0	18.0
2010													52.8	21.8				
2011							46.6	34.2	17.5	40.3	28.0	12.7	53.1	22.2				
2012													53.5	22.6				
2013													53.6	22.8				
2014													54.1	23.3				

*Source and Note*: HMRC Series C – table 13.5 on the HMRC website at http://webarchive.nationalarchives.gov.uk/20120403124426/ http://www.hmrc.gov.uk/stats/personal_wealth/13‐5‐table‐2005.pdf.

Derived from HMRC new series – estimated from table 13.1 on the HMRC website at https://www.gov.uk/government/uploads/system/uploads/attachment_data/file/447352/table_13‐1.pdf.

ONS WAS for OECD – estimates provided by the ONS to the OECD Wealth Database.

Credit Suisse – Credit Suisse Research Institute, 2014.

Vermeulen – Vermeulen, 2015. Vermeulen's top wealth shares derived uniquely from the WAS (before any combination with the Forbes Rich List) are 30 per cent for the top 5 per cent and 13 per cent for the top 1 per cent.

Series C ended in 2005. HM Revenue & Customs (2012) explains the main changes in methodology in the new estimates, replacing Series C. The most important are the move to producing estimates based on data averaged over three years, the most recent being 2008–10, in order to reduce sampling variation, and the adoption of new multipliers based on the variation of

mortality with housing wealth.^25^ On the other hand, HMRC has dropped the adjustments made in Series C for the excluded wealth, for valuation and to a balance‐sheet basis, focusing exclusively on the identified wealth. The link with the National Accounts balance sheets has been broken, making very difficult the estimation of the total personal wealth for these years. HMRC's reasons for dropping these adjustments are described as follows:

These adjustments were not based on robust data, and used operational adjustments or assumptions instead. We do not know how accurate these adjustments are or if they should be changing over time. The data on Adjusted and Marketable Wealth is sensitive to these assumptions and so it was decided that this data was not robust enough for us to continue to publish it.^26^

These concerns are understandable. For example, the Board of Inland Revenue (2000) describes how the estimate of excluded wealth in trusts was based on studies for two years (1976 and 1988) which were by then distant in time. Although small in total, the addition would be largely allocated to the upper wealth groups. A significant investment would no doubt have been required to bring the estimate up to date. It is, however, regrettable that such an investment, and investments in other elements, were not given priority and that, as a result, the estimates of the wealth distribution are now less complete.

The ‘new HMRC estimates’^27^ show the numbers and total wealth of individuals by ranges of net unadjusted wealth. In particular, the estimated wealth is not corrected for potential under‐reporting and undervaluation as was done for Series C data. The same data are also presented in the form of decile shares, but these are of little interest since they relate only to those identified as wealth‐holders (in 2008–10, only 31 per cent of the total population aged 18 and over) and only to identified wealth. In order to render the estimates closer to those for earlier years, we have made two adjustments. First, we have expressed the numbers as a percentage of the total population aged 18 and over. Second, we have taken as the control total for wealth the sum of identified wealth plus excluded wealth as estimated by HMRC,^28^ where this includes an estimate of the wealth of the excluded population. This procedure is applied up to 2005, the last year for which the HMRC reconciliation exercise has been published, and for subsequent years is extrapolated in line with total personal wealth as estimated in the UK balance sheet.^29^ The results are the estimates ‘derived from HMRC new series’ shown in Table 1.

Comparing the estimates in Figures 2 and 3 for the overlapping period 2001–03 (with data points for the new ones plotted at 2002), we can see that the new HMRC estimates are, as we would expect, lower than the earlier Series C estimates. The share of the top 1 per cent is 3.1 percentage points lower. It should be noted that there is a considerable margin of error around our estimated control total. Series C, indeed, cannot be directly compared to the new assembled series from HMRC due to the lack of adjustments for wealth valuations.

What do the estate‐based estimates tell us about the three questions with which we began? First, they indicate that the distribution of wealth in the UK is highly concentrated. The top 1 per cent own between one‐fifth and one‐quarter of total personal wealth. For instance, adding 3.1 percentage points to the estimate for 2008–10 gives a figure roughly comparable to those for Series C of 23.5 per cent for the share of the top 1 per cent.

Second, if we take the estimates in Table 1, then the share of the top 1 per cent in total net worth (of individuals) is around double the share of the top 1 per cent (again, of individuals) in total net income (income after deducting income tax), which in the first half of the 2000s was around 10 per cent.^30^ The share of the top 10 per cent in total wealth was at that time about 50 per cent higher than the share of the top 10 per cent in total net income. Of course, the top *x* per cent of wealth‐holders are not necessarily the same people as the top *x* per cent of income‐recipients.

Third, there is some indication that the top shares in wealth were increasing between 2001–03 and 2008–10, but this may depend on the estimation of the wealth control total, which is now subject to higher uncertainty as explained above. We would therefore be cautious about drawing any firm conclusion in view of the need for a more robustly established control total for wealth.

### 2. Household‐survey‐based estimates

The introduction of the Wealth and Assets Survey provides a new and independent source of evidence about the distribution of wealth in Great Britain. Figures 2 and 3 show the estimated shares of the top 10 and 1 per cent as supplied to the OECD by the ONS for the three periods covered (in each case shown at the mid‐point year – for example, 2007 for 2006–08). These shares relate to household wealth (each household weighted as 1), and are shown including and excluding pension rights (the latter data for 2010–12 are included in the OECD Wealth Distribution Database, labelled 2012).

The first finding is that, in the case of the overlapping period 2008–10, the WAS estimates excluding pension wealth suggest a share of the top 10 or top 1 per cent that is considerably below the estate‐based estimates: 43 per cent for the top 10 per cent, compared with 54 per cent, and 14 per cent for the top 1 per cent, compared with 20 per cent. We have to take account of the fact that these estimates are household‐based and that the geographic coverage differs, but the difference is larger than could be explained in this way. Moreover, if pension wealth is included, then the gap is even wider. The share of the top 1 per cent is only 11 per cent, or virtually half that found in the estate‐based estimates. If the share of the top 1 per cent were as low as 11 per cent, then we would have to revisit the conclusion that wealth is much more unequally distributed than income: the share of the top 1 per cent in after‐tax income in 2009–10 averaged 10.7 per cent.

These estimates for Great Britain are compared by the OECD with estimates for other countries based on sample surveys. It is interesting to begin with an earlier such comparison: that between Great Britain and the United States based on household surveys in the 1950s.^31^ This found that the distribution of wealth was significantly more unequal in Britain. Sixty years later, the OECD figures show that the reverse is the case: the share of the top 1 per cent in the US in 2010 is 36.6 per cent, or more than double the UK figure for 2009.^32^ This dramatic change warrants further investigation, as does the fact that the top 1 per cent wealth share in Great Britain is so much lower (even leaving aside pensions) than in Austria and the Netherlands (both 24 per cent) and Germany (25 per cent).

The second finding is that the WAS‐based estimates supplied by the ONS to the OECD show a distinct upward trend. The share of the top 1 per cent in 2010–12 is 2.7 percentage points higher than that in 2006–08, when measured including pension wealth, and the increase is nearly double (5.3 percentage points) for the estimates excluding pension wealth. Such a striking conclusion also needs to be investigated further.

### 3. Combined with the Rich Lists

The OECD (2015) refers to the problems with studying the upper tail of the wealth distribution using household surveys: ‘measuring wealth at the top of the wealth distribution through household surveys is intrinsically difficult, as wealthy households typically under‐report their wealth [and] household surveys suffer from varying degrees of non‐response [– the] bias is particularly large when looking at the top 1 per cent of the distribution’ (p. 251). This has led to attempts to use independent data from Rich Lists to ‘complete’ the survey data. Vermeulen (2015) has combined extreme wealth observations from the Forbes List of World Billionaires with the WAS data for 2008–10. He begins by noting ‘there is a substantial gap between the highest ranked survey household and the lowest ranked Forbes individual’ (p. 18). Fitting a Pareto upper tail, he finds that the share of the top 1 per cent rises by between 1 and 5 percentage points, depending on the threshold assumed for the Pareto distribution.^33^ The higher end of this range would go some way towards closing the gap between the household survey estimates and those for individual wealth‐holding based on the estate data for 2008–10. At the same time, we should note that those identified in the Forbes List may include people who are not UK residents.

A Pareto extrapolation had been used earlier by Davies and Shorrocks in the estimates they have prepared for Credit Suisse.^34^ In effect, they use the total number of billionaires (but not their wealth) reported in the Forbes List to fit a Pareto distribution. It is the changing number of billionaires that drives the year‐to‐year changes shown in Figures 2 and 3 (the dashed series), since the distribution is otherwise based on the WAS 2006–08. As may be seen, these estimates suggest that the share of the top 1 per cent is close to the estate‐based estimates, and the share has increased by some 3 percentage points over the period from 2000 to 2014.

The Rich Lists provide information on the shape of the upper tail of the wealth distribution that allows for a more detailed investigation of the distribution within the top 1 per cent. To date, official estimates of wealth concentration have not shown shares for groups smaller than the top 1 per cent (the same limitation applied to the findings in Atkinson and Harrison (1978)). The Sunday Times Rich List for 2010 headline has 1,000 people with £335.5 billion. These make up 0.004 per cent of total (GB) households and 5.3 per cent of total WAS non‐pension wealth.

## V. Conclusions

In this paper, we have used evidence from existing data sources to attempt to answer the three questions set out at the beginning and to identify the need for further information. The UK wealth distribution is indeed highly concentrated. The estate‐based estimates (the former HMRC Series C, the unadjusted estimates and the new HMRC estimates, allowing for the understatement of concentration) suggest that the share of the top 1 per cent is between a fifth and a quarter of total personal wealth. The household survey data cannot be used on their own to investigate concentration at the top. When combined with information about the upper tail, the survey‐based estimates (excluding pension wealth) are below the estate‐based estimates of top shares, but we have to allow for the fact that the estimates relate to households rather than individuals. On the basis of the estate‐based estimates, wealth inequality at the top exceeds inequality in after‐tax income: the share of the top 1 per cent in total wealth is about double the share of the top 1 per cent in after‐tax income. Finally, the estimates provide some support for the view that wealth inequality increased in the UK over the first decade of the present century, but we believe that any definitive statement should await further investigation.

Indeed, the evidence about the UK distribution of wealth post‐2000 is seriously incomplete and the main conclusion of the paper is that significant investment is necessary if we are to provide satisfactory answers to the three questions. Moreover, given the limitations of each of the different sources, it is important to make use of all available approaches. The estate‐based estimates remain, in our view, an essential element when studying top wealth‐holdings (and we do not believe that the HMRC official estimates should be discontinued as currently proposed), but there needs to be a renewed investigation of the mortality multipliers, the necessary adjustments and the reconciliation with the balance‐sheet information. The investment income method should be explored further, but for this it is necessary that the underlying data be available in a more detailed form. The issues of non‐response and under‐reporting at the top mean that the household surveys – valuable though they are in covering the majority of the population – need to be supplemented when considering the upper tail. Consideration needs to be given to the use of investment income data for this purpose, in addition to the Rich Lists. These recommendations require resources, but unless such work is undertaken we shall not be able to draw firm conclusions about the extent of wealth concentration, how it compares with that in other countries and whether it is increasing over time.

## Supporting information

Disclaimer: Supplementary materials have been peer‐reviewed but not copyedited.

Appendix

## Footnotes

1 OECD, 2015, p. 34.

2 OECD, 2015, p. 241.

3 There exists a fifth potential source of evidence about the distribution of personal wealth: administrative data on the wealth of the living derived from personal wealth taxes. However, in the UK there is no annual wealth tax, and the council tax cannot be used for this purpose.

4 Namely, the estates gaining a grant of representation (known as confirmation of executors in Scotland, and probate or letter of administration in the rest of the UK). The estates held by younger individuals are oversampled, while 100 per cent of the largest estates are included. With this procedure, estates of low value are generally excluded, as well as those held in trusts or in joint names passing to a surviving spouse or civil partner. The excluded estates accounted for approximately 70 per cent of total estates in 2008–10.

5 Available from the HMRC website at http://webarchive.nationalarchives.gov.uk/20120403124426/ http://www.hmrc.gov.uk/stats/personal_wealth/archive.htm (Series C) and https://www.gov.uk/government/collections/distribution‐of‐personal‐wealth‐statistics (new series).

6 Although, for many years, the Inland Revenue made estimates of the distribution of wealth including occupational pension rights (Series D) or both occupational and state pension rights (Series E) – see, for example, *Inland Revenue Statistics 1996*, tables 13.6 and 13.7.

7 See Atkinson and Harrison (1978, pp. 32–3).

8 This represents an improvement on the ground that homeownership can hardly identify wealth in a context of high and increasing homeownership rates. However, as acknowledged by HMRC, the data still present some problems to the extent that ELSA is designed to be representative of older households living in England only.

9 See Atkinson and Harrison (1978, p. 175).

10 This explains why, at this stage, we do not provide results based on the investment income method in Section 3..

11 Table 13.1 on the HMRC website at http://webarchive.nationalarchives.gov.uk/20120403124426/ http://www.hmrc.gov.uk/stats/personal_wealth/archive.htm.

12 See Atkinson and Harrison (1978, appendix I).

13 Royal Commission on the Distribution of Income and Wealth, 1979, p. 117.

14 Office for National Statistics, 2012.

15 Office for National Statistics, 2009, p. 2.

16 Office for National Statistics, 2009, p. 119.

17 Office for National Statistics, 2009, p. 5.

18 Beresford, 1990, 1991 and 2006.

19 For example, page 91 of the *Sunday Times Magazine*, 18 May 2014.

20 Dolan, 2012.

21 Table 13.4 on the HMRC website at http://webarchive.nationalarchives.gov.uk/20120403124426/ http://www.hmrc.gov.uk/stats/personal_wealth/archive.htm.

22 This may clearly vary across countries depending on the nature of the tax unit. In the US, for instance, a tax unit is similar to a family unit as it contains singles or married couples with or without dependants. Therefore the estimates of top wealth shares for the US by Saez and Zucman (2014) based on the capitalisation method relate to tax units and not to individuals.

23 Notwithstanding this, the WAS aims to follow individuals rather than households. In the case that a household splits, with individuals living at different addresses, WAS interviews all of the original sample members in the next wave of the survey (Office for National Statistics, 2014).

24
http://www.ons.gov.uk/ons/rel/cap‐stock/the‐national‐balance‐sheet/2014‐estimates/index.html.

25 The next update, covering the period 2011–13, was scheduled for publication in September 2015. However, as we write, HMRC is proposing not to publish this or any further updates, the reason being that it ‘[does] not think that the HMRC Personal Wealth National Statistics, which are based on data from Inheritance Tax returns for estates requiring probate, can be reliably used to look at the distribution of wealth amongst all people in the UK’ (HM Revenue & Customs, 2015, p. 4). There are indeed limitations to the estate‐based estimates, as we have noted above and as has been extensively discussed in the literature over more than a century (a literature to which the Inland Revenue, the predecessor of HMRC, has been a major contributor). But, as we have emphasised, other sources of data on wealth‐holding have significant limitations regarding the coverage of top wealth‐holders.

26 HM Revenue & Customs, 2012, p. 17.

27 Table 13.1 on the HMRC website at https://www.gov.uk/government/uploads/system/uploads/attachment_data/file/447352/table_13‐1.pdf.

28 Table 13.4 on the HMRC website at http://webarchive.nationalarchives.gov.uk/20120403124426/http:/www.hmrc.gov.uk/stats/personal_wealth/archive.htm.

29 Blue Book 2014, S1HN‐LE‐B90: total net worth of households and NPISH.

30 The shares of the top 10, top 5 and top 1 per cent of net income are provided in the online appendix. The numbers are different from those appearing in the World Top Incomes Database (WTID) simply due to the different definition of the population control total: adults aged 18 and over in this paper (for consistency with the wealth distribution estimates), and adults aged 15 and over in the WTID.

31 Lydall and Lansing, 1959.

32 Cowell (2013, p. 44) draws attention to the ‘surprising’ finding from the earlier BHPS survey in 2000 that the UK exhibits less wealth inequality than the US, Canada and Sweden.

33 See also Cowell (2013) on fitting a Pareto distribution to household survey data – in this case, data from the Luxembourg Wealth Study.

34 See, for example, Credit Suisse Research Institute (2014, p. 9), which describes the way in which their method has changed over time.
