# Supplementary material for: The Challenge of Measuring UK Wealth Inequality in the 2000s
Source: Fisc Stud. 2016 Mar 31;37(1):13–33. doi: 10.1111/j.1475-5890.2016.12084 (PMC5001192; doi:10.1111/j.1475-5890.2016.12084)
Supplement: Supplementary file 1 — Appendix [file FISC-37-13-s001.pdf]

Appendix

	Top 1% income share - net of income tax			Adult population	Wealth aggregates							
	top 10%	top 5%	top 1%	aged 18 years and over	Identified wealth	Wealth adjustment for under-recording and differences in valuation	Excluded wealth	Identified plus excluded wealth	HMRC Series C marketable wealth	National Accounts balance sheet for S.14 and S.15	Ratio marketable/balance-sheet wealth	Wealth total used for the estimates derived from HMRC new series
	%	%	%	thousand	billion £	billion £	billion £	billion £	billion £	billion £	%	billion £
	[1]	[2]	[3]	[4]	[5]	[6]	[7]	[8]=[5]+[7]	[9]=[5]+[6]+[7]	[10]	[11]=100*[9]/[10]	[12]
2000	36.1	24.3	10.6	45,480	2,201	183	738	2,939	3,122	4,822	64.8	2,939
2001	36.4	24.3	10.5	45,756	2,481	181	802	3,283	3,464	4,792	72.3	3,283
2002	36.0	23.9	10.2	46,048	2,623	222	846	3,469	3,691	5,127	72.0	3,469
2003	36.4	24.3	10.4	46,354	2,839	263	948	3,787	4,050	5,503	73.6	3,787
2004	36.0	24.1	10.5	46,689						5,962		
2005	36.4	24.8	11.2	47,163	3,432	665	908	4,340	5,005	6,376	78.5	4,340
2006	36.8	25.4	11.8	47,592						6,771		4,634
2007	37.4	26.1	12.3	48,043						7,204		4,931
2008				48,499						6,574		4,500
2009	36.5	25.3	12.2	48,910						6,968		4,769
2010	32.7	21.9	9.2	49,371						7,517		5,145
2011	33.5	22.5	9.4	49,839						7,906		5,411
2012	33.1	22.2	9.3	50,180						8,240		5,640
